# Screening and Characterization of Some *Lactobacillaceae* for Detection of Cholesterol-Lowering Activities

**DOI:** 10.1007/s12602-022-09959-9

**Published:** 2022-06-15

**Authors:** Martin Frappier, Julie Auclair, Samir Bouasker, Sathursha Gunaratnam, Carine Diarra, Mathieu Millette

**Affiliations:** Bio-K Plus International Inc., a Kerry Company, 495 Armand-Frappier Boulevard, Laval, QC H7V 4B3 Canada

**Keywords:** Bile salt hydrolase, Feruloyl esterase, Probiotic, Cholesterol, *Lactobacillus acidophilus* CL1285, *Lactiplantibacillus plantarum* CHOL-200

## Abstract

Dyslipidemia, specifically abnormal levels of low-density lipoprotein cholesterol (LDL-C), is an important risk factor of cardiovascular disease. Evidence showing the promising abilities of probiotics to lower total cholesterol or LDL-C has, however, not yet convinced experts to recommend probiotic bacteria as treatment for blood lipid management. Therefore, there are opportunities for the development of new efficient cholesterol-lowering probiotics. Bile salt hydrolase (BSH) and feruloyl esterase (FAE) are bacterial enzymes proposed to explain the cholesterol-lowering capacity of some bacteria and have both been shown to be responsible for lipid reduction in vivo. Here, in order to select for cholesterol-lowering bacteria, 70 strains related to *Lactobacillaceae* were screened for BSH and FAE activities. Based on this two-way screening approach, two bacteria were selected and assessed for their capacity to assimilate cholesterol in vitro, another suggested mechanism. *Lactobacillus acidophilus* CL1285 showed BSH and FAE activity as well as capacity to assimilate cholesterol in vitro. *Lactiplantibacillus plantarum* CHOL-200 exhibited BSH activity and ability to assimilate cholesterol. These properties observed in vitro make both strains good probiotic candidates for the management of dyslipidemia. Further investigation is needed to assess their ability to reduce blood cholesterol in human trial.

## Introduction

Cardiovascular disease (CVD) is the leading cause of death worldwide [1]. Dyslipidemia, abnormal levels of blood lipids, especially low-density lipoprotein cholesterol (LDL-C), is a major risk factor for CVD [2, 3]. Countless pharmaceutical drugs are available by prescription and are used all around the world to normalize serum lipids in hypercholesterolemic patients. However, the vast majority of these drugs cause adverse events such as muscular pain, diabetes, decrease in renal function, and an increase in depression-like symptoms [4]. Over 30% of all patients using statins experience muscle pain [5]. These side effects lead many patients to seek alternative solutions to improve their lipid metabolism, such as the utilization of various foods or supplements like green tea, soluble fiber, or garlic [6, 7]. Additionally, probiotic bacteria have gained much attention due to their strain-specific cholesterol-lowering effects [8].

Probiotics are defined as live microorganisms that, when administered in adequate amounts, confer a health benefit on the host [9]. *Lactobacillaceae* are well-characterized probiotic bacteria that show promising cholesterol-lowering capacity in vitro as well as in animal and clinical trials [10, 11]. In a meta-analysis, Wu et al. analyzed the results of 15 randomized human clinical trials to determine the effect of consumption of *Lactobacillus* spp. on the serum lipid profile [12]. By pooling 14 of these studies, the authors showed that *Lactobacillus* spp. could significantly reduce blood levels of total cholesterol (TC) and LDL-C. Subgroup analysis highlighted the potential of *Lactiplantibacillus* (*Lactobacillus*) *plantarum* and *Limosilactobacillus* (*Lactobacillus*) *reuteri* to significantly lower TC and LDL-C. Also, Sun and Buys revealed, based on the pooled effect of 10 clinical studies, that consumption of probiotics, especially formulations containing multiple strains and those containing *Lactobacillus acidophilus*, was effective to decrease LDL-C levels [13].

Among potential mechanisms that have been proposed to explain the cholesterol-lowering activity observed with some probiotic bacteria, the most well studied are bile salt hydrolase (BSH) activity, assimilation of cholesterol, conversion of cholesterol to coprostanol, co-precipitation of cholesterol with unconjugated bile salts, feruloyl esterase (FAE) activity, and modulation of NPC1L1 gene expression [14–16]. BSH, synthesized by some bacteria related to *Lactobacillaceae*, *Bifidobacterium* spp., *Enterococcus* spp., *Clostridium* spp., and *Bacteroides* spp., breaks the peptide linkage between primary bile acid and either glycine or taurine and liberates the amino acid from the sterol core [17–21]. The unconjugated bile acids released by the enzyme are less soluble and stay in the intestinal lumen. These unconjugated bile acids are mixed with intestinal content and naturally excreted in feces. This mechanism thus forces the body to synthesize new bile acid molecules de novo with cholesterol present in the host, thereby reducing circulating blood cholesterol [22, 23].

Another suggested mechanism is feruloyl esterase (FAE) activity. This enzyme has been found in some lactic acid bacteria (LAB) such as *Lactobacillus gasseri*, *Lactobacillus acidophilus*, *Lactobacillus helveticus*, *Lactobacillus johnsonii*, and *Limosilactobacillus* (*Lactobacillus*) *fermentum* and is involved in the liberation of phenolic compounds, such as ferulic acid (FA), from plant cell walls [24–26]. FA can act as a competitive inhibitor of one of the main enzymes responsible for de novo synthesis of cholesterol, 3-hydroxy-3-methylglutaryl-coenzyme A reductase (HMG-CoA reductase), the same target as statins [27]. FA can also activate the *peroxisome proliferator-activated receptor* alpha (PPARα), a nuclear transcription factor implicated in the synthesis of two-cholesterol transporters, high-density lipoprotein (HDL), and LDL. PPARα activation has been associated with an increase of HDL and a decrease of LDL [28].

Moreover, the strain-specific capacity of certain LAB to assimilate cholesterol in vitro has been investigated. Cholesterol incorporated into the cell membrane of bacteria, which are excreted through feces, could decrease the overall pool of circulating blood cholesterol [23, 29–31].

Bio-K Plus International Inc. (Bio-K +) is specialized in the development and manufacturing of a lactobacilli-based probiotic formulation, combining the *Lactobacillus acidophilus* CL1285, *Lacticaseibacillus* (*Lactobacillus*) *casei* LBC80R, and *Lacticaseibacillus* (*Lactobacillus*) *rhamnosus* CLR2 strains. This formulation has previously showed to significantly reduce the incidence of antibiotic-associated diarrhea and *Clostridioides* (*Clostridium*) *difficile* infections and improve the quality of life in people with diarrhea-predominant irritable bowel syndrome [32–36]. In this study, a Bio-K + proprietary collection of *Lactobacillaceae* has been screened using a multi-mechanism approach, targeting BSH and FAE activities, to select bacterial candidates with the potential to manage and reduce cholesterol levels in humans. Following screening, *L. acidophilus* CL1285 and *L. plantarum* CHOL-200 were selected based on their strong BSH and FAE activities and further characterized for their capacity to assimilate cholesterol in vitro and for their probiotic properties.

## Experimental Section

### Bacteria Used in This Study

All bacteria were grown on de Man, Rogosa, Sharpe (MRS) agar (EMD Millipore, No. 1.10661) under anaerobic atmosphere using a Bactron300 anaerobic chamber (Sheldon Manufacturing Inc., Cornelius, OR, USA) containing a gas mixture made of 5% hydrogen, 10% nitrogen, and 85% carbon dioxide (MEGS, a division of Air Liquide, Montreal, Canada). BK strains, as well as strains *L. acidophilus* CL1285, *L. casei* LBC80R, and *L. rhamnosus* CLR2, are Bio-K + ’s proprietary strains and have been isolated from food or from human milk, oral, vaginal, and fecal samples. Other strains were purchased from the American Type Culture Collection (ATCC, Manassas, VA, USA), the Belgian Coordinated Collections of Microorganisms (BCCM/LMG, Gent, Belgium), and the Patent and Bio-Resource Centre (Formerly Fermentation Research Institute; FERM, Chiba, Japan). For human bacterial isolates, participants were informed of the purpose of the study and gave their informed consent before providing self-collected samples. Bacteria were stored at − 80 °C and thawed at room temperature before use. Before each experiment, bacteria were streaked on MRS plates to verify their purity.

### Bacterial Identification

Bacterial identification was performed by sequencing the *rrs* gene coding for the 16S ribosomal RNA (rRNA) (Génome Québec, Montreal, Canada). Sequences were then compared to homologous bacterial sequences using BLAST (www.ncbi.nlm.nih.gov/BLAST). For DNA extraction, bacterial colonies were resuspended in a 2-mL sample tube containing 100 µL of 10 mM Tris–HCL (Fisher Scientific, No. BP153) and 100 mg of glass beads (Sigma-Aldrich, N. Z250465). The samples were placed horizontally on a vortex, using a vortex adapter, and were bead beaten at maximum speed for 2 min. Samples were cooled on ice for 2 min. The bead beating and cooling steps were repeated twice and the samples were centrifuged at 10,000 × g for 2 min. Supernatants were then used for DNA amplification. DNA coding for 16S rRNA gene sequences was amplified in a DNA thermal cycler (Tpersonal Thermocycler, Biometra, Goettingen, Germany) using the pA (5′-AGAGTTTGATCCTGGCTCAG-3′) and pH (5′-AAGGAGGTGATCCAGCCGCA-3′) primers [37]. PCR amplifications were performed in a 50 µL reaction volume with 20 µg bovine serum albumin (Sigma-Aldrich, No. B8667), 10 pmol of each primer (AlphaDNA, Montreal, Canada), 4 µL of DNA sample, and 2.5 U of MyTaq™ HS DNA polymerase (Bioline, No. Bio-21113) in 1X MyTaq™ buffer. The amplifications were performed at 95 °C for 5 min and 55 °C for 5 min; then 30 cycles at 72 °C for 45 s, 94 °C for 45 s, and 55 °C for 45 s; and a single final extension step at 72 °C for 10 min [38]. Table 1 lists all bacterial genera used in this study. 16S rRNA gene sequences of *L. acidophilus* CL1285 and *L. plantarum* CHOL-200 have been deposited on GenBank and can be accessed via accession numbers ON544019 and ON544020.

**Table 1:** Number of each bacterial genus of *Lactobacillaceae* used for screening purposes in this study

Genus	Number of bacteria
*Lacticaseibacillus*	15
*Lactiplantibacillus*	14
*Lactobacillus*	17
*Lentilactobacillus*	7
*Levilactobacillus*	10
*Limosilactobacillus*	7
**Total**	**70**

### Detection of BSH Activity

Evaluation of BSH activity was conducted using a method described by Pereira et al. [39]. A loop of fresh bacterial culture was streaked on the surface of MRS agar (1.5% w/v) (EMD Millipore, No. 1.10661) supplemented with 0.5% sodium taurocholate hydrate (TCA) (Sigma-Aldrich; No. 86339) or 0.5% sodium glycocholate hydrate (GCA) (Sigma-Aldrich, No. G7132) and 0.37 g/L calcium chloride anhydrous (Labo MAT, No. CP-0108). Each plate was incubated under anaerobic atmosphere at 37 °C for 72 h. BSH activity was characterized by the presence of a precipitate around the colony, a rough aspect of the colony, or both. Semi-quantitative data are expressed by “ + ” symbol comparing the growth and/or precipitation zone of each bacteria strain on an agar plate with and without bile salts. The number of “ + ” symbols is proportional to the intensity of the activity. A negative result is indicated by a negative symbol, meaning the bacteria can grow on the medium but no activity was detected when compared with the same medium without bile salts.

### Detection of FAE Activity

Detection of FAE activity was performed using a semi-quantitative method described by Donaghy et al. with some modifications [40]. Briefly, a modified MRS medium with 1.5% agar (Fisher Scientific, No. BP1423) was prepared using all the same ingredients for the MRS medium broth, but without glucose. The medium was sterilized at 121 °C for 20 min and cooled at 50–55 °C. Six milliliters of a solution containing 10% ethyl 4-hydroxy-3-methoxycinnamate (ethyl ferulate) (Sigma-Aldrich, No. 320617) dissolved in N,N-dimethylformamide (Sigma-Aldrich, No. D4551) was added to 500 mL of medium and gently mixed. The medium was then poured into sterile Petri dishes and solidified at room temperature. A loop of fresh bacterial culture was streaked on the surface of each Petri dish and the plates were incubated under anaerobic atmosphere at 37 °C for 3 to 6 days. All results are expressed by comparing the growth of each strain and disappearance of the fogginess around each bacterial colony on an agar plate with and without ethyl ferulate. Plus “ + ” symbols were used to describe FAE activity; the number of symbols is proportional to the FAE activity. A negative result is indicated by a negative symbol, meaning the bacteria can grow on the medium, but no activity was detected when compared with the same medium without ethyl ferulate.

### Cholesterol Assimilation by the LAB

Cholesterol micelles were prepared according to the method developed by Razin et al. with some modifications [41]. Briefly, 100 mg of cholesterol (Sigma-Aldrich, No. C8667) and 220 mg of lecithin soybean—a mixture of phosphatidylcholine and lysolecithin (American Lecithin Company, No. LPC50)—were solubilized in 10 mL of chloroform (Fisher Scientific, No. BP1145). Chloroform was evaporated using nitrogen gas (Praxair Canada Inc., Laval, Quebec). Then, 30 mL of a 0.4 M sucrose solution (Fisher Scientific, No. S5) was added and the cholesterol/lecithin mixture was sonicated to produce cholesterol micelles. Sonication was performed on ice using a Sonic Dismembrator 500 (Fisher Scientific, Pittsburg, PA, USA) at maximum amplitude with power set between 100 and 120 watts for three periods of 15 min, renewing ice between each period. The solution was then centrifuged (Avanti J-26S XPI, Beckman Coulter, Brea, CA, USA) in high-speed centrifuge tubes for 30 min at 30,000 × g to eliminate metal particles from the sonicator probe.

After two successive overnight anaerobic incubations in MRS broth, each culture was washed using saline water (NaCl 0.85% w/v) and pellets were resuspended in sterile water. Then, 100 µL of the pellet suspension was mixed with 1 mL of cholesterol micelles in pre-reduced MRS medium supplemented with 0.2% sodium thioglycolate (Sigma-Aldrich, No. T0632) and 0.6% Ox gall (Sigma-Aldrich, No. B3883). Subsequently, all tubes were incubated under anaerobic atmosphere at 37 °C for 48 h. The strain *L. acidophilus* ATCC 43121 was selected as positive control.

Quantification of cholesterol assimilation by bacteria was measured using the method described by Rudel and Morris and Gilliland et al. with some modifications [29, 42]. Briefly, the contents of each tube, containing 9 mL of culture, were centrifuged at 3,700 × g for 15 min and the pellets, containing the assimilated cholesterol, were resuspended with the same volume of sterile water. One milliliter of each sample was transferred to a borosilicate tube containing 3 mL of 95% ethanol (Les Alcools de Commerce, No. USP + 432,526) and 2 mL of a 50% potassium hydroxide solution (Acros Organics, No. 232550010). Each tube was vortexed 20 s and then heated in a water bath at 60 °C for 10 min. Then, the tubes were cooled at room temperature for 10 min. Five milliliters of *n*-hexane (Acros Organics, No. AC160780010) and 3 mL of sterile distilled water were subsequently added to the tubes and vortexed for 30 s. The tubes were incubated at room temperature for 15 min to allow phase separation. After that, 2.5 mL of the superior phase was transferred to another borosilicate tube. The tube was warmed in water heated at 70 °C and *n*-hexane was completely evaporated using nitrogen gas. The pellet was resuspended in 4 mL of a freshly prepared solution of *o*-phthalaldehyde (Sigma-Aldrich, No. 79760) at a concentration of 0.5 mg/mL previously dissolved in glacial acetic acid (Fisher Scientific, No. 351271). Tubes were incubated at room temperature for 10 min. Then, 2 mL concentrated sulfuric acid (Fisher Scientific, No. 351298–212) was added to each tube, mixed for 20 s by vortexing, and again incubated for 10 min at room temperature. OD_550nm_ of each sample was measured using a BioPhotometer plus (Eppendorf, Hamburg, Germany). Blank consisted of 4 mL of *o*-phthalaldehyde solution and 2 mL of concentrated sulfuric acid. Standard curve was generated using a cholesterol solution of 0, 25, 50, 75, 100, 200, and 400 μg/mL. TC was determined by subtracting the OD_550nm_ of thioglycolate- and bile-supplemented MRS medium without cholesterol from the OD_550nm_ of thioglycolate- and bile-supplemented MRS media containing cholesterol micelles. All standards and samples were subjected to identical procedures. Assimilation percentage was calculated using the following formula:

$$\mathrm{\% assimilation}=\left(\frac{\mathrm{Cholesterol\ from \ pellet }\left(\mathrm{\mu g}/\mathrm{mL}\right)}{\mathrm{Total \ cholesterol }\left(\mathrm{\mu g}/\mathrm{mL}\right)}\right)\times 100$$

For each bacterium, percentage of cholesterol assimilation was evaluated from three independent samples run in duplicate. Values are expressed as mean ± standard deviation. Data were analyzed by one-way analysis of variance (ANOVA), using SPSS Statistics for Windows, version 19.0 software (IBM Corporation, Armonk, NY, USA). Differences among the groups were analyzed with a post hoc Duncan’s multiple range test. Differences between means were considered significant at *p* ≤ 0.05 [43].

### Gastric Survival

On the day of the experiment, simulated gastric fluids (SGF) pH 1.5, 2, and 2.5 were prepared. Briefly, 2 g of sodium chloride (Fisher Scientific, No. BP358-212) and 3.2 g of pepsin (Sigma-Aldrich, No. P7000) were dissolved in 900 mL of osmosis filtered water. The pH was adjusted with 5 M hydrochloric acid (Fisher Scientific, No. A144-212) and measured using an Orion 3 Star pH BenchTop (Thermo Scientific, Beverly, MA, USA) equipped with a Ross Ultra pH/ATC Triode. Following pH adjustment, SGF volume was brought up to 1 L using sterile osmosis water and 19 mL of SGF was transferred to 50-mL conical tubes. Before the experiment, each tube was placed in an incubator at 37 °C for 1 h to warm the solution.

One milliliter of an overnight culture of *L. acidophilus* CL1285 or *L. plantarum* CHOL-200 was added to each tube containing SGF and was immediately placed into a MaxQ 4450 Incubator-shaker (Thermo Scientific, Marietta, OH, USA) preheated to 37 °C and agitated at 250 rpm for 30 and 60 min. After incubation, 1 mL of each SGF-bacterial mixture was neutralized in 9 mL of a sterile solution of 0.1 M sodium phosphate buffer. Bacteria were enumerated by ten-fold serial dilution in peptone water (1 g/L peptone and 0.5 g/L sodium chloride). Appropriate dilutions were plated on MRS agar (1.5% w/v) and incubated under aerobic atmosphere at 37 °C for 48 h.

### Bile Salt Tolerance

Bile salt tolerance was performed on both *L. acidophilus* CL1285 and *L. plantarum* CHOL-200 strains, using three different types of bile. Tolerance to Ox gall powder (Sigma-Aldrich, No. B3883, Bile Salts (Sigma-Aldrich, No. 48305) and bacteriological bile (OrganoTechnie, No. 10236) was evaluated by inoculating the bacteria on plates containing either 0.15% or 0.3% (w/v) of each type of bile. Each strain was streaked on the surface of a bile salt-supplemented MRS agar plate and incubated under anaerobic atmosphere at 37 °C for 48 h. Growth on MRS agar without bile salts was used as a positive control to determine the capacity of each strain to grow on each type of supplemented MRS agar.

## Results

A total of 70 bacteria belonging to the family *Lactobacillaceae*, isolated from human feces, oral, vaginal and milk samples, foods, or purchased from commercial suppliers, were screened for the presence of BSH or FAE activities. Of these, 18 harbored BSH activity, with 10 strains being apparently selective to the taurine-conjugated form of bile acids (TCA), and five exhibiting specific activity toward glycine-conjugated bile acids (GCA). In addition, three strains related to *Lactobacillus acidophilus*, *Lacticaseibacillus casei*, and *Lactiplantibacillus plantarum* showed the capacity to deconjugate bile acid with either taurine or glycine. Among these three strains, two appear to have stronger BSH activity. *L. acidophilus* CL1285 and *L. plantarum* CHOL-200 exhibited, in the presence of either GCA or TCA, robust BSH activity compared to all other *Lactobacillaceae* tested (Table 2).

**Table 2:** Bile salt hydrolase (BSH) and feruloyl esterase (FAE) activities. -: negative, + : positive, +  + : strongly positive

Bacteria	Strain origin	BSH		FAE
		**GCA**^**a**^	**TCA**^**b**^	
*L. casei*				
BK16	Human oral mucosa	-	-	-
BK26	Human oral mucosa	-	-	-
BK28	Human oral mucosa	-	-	-
BK35	Human oral mucosa	-	-	-
LBC80R	Human fecal microbiome	+	+	-
ATCC 393	Dairy	-	+ +	-
*L. paracasei*				
BK1	Human oral mucosa	-	-	-
BK20	Human oral mucosa	-	-	-
BK91	Human oral mucosa	-	-	-
BKLM.4	Human milk	-	-	-
BK86	Human fecal microbiome	-	-	-
BKJA.1	Human fecal microbiome	-	-	-
BK84	Human fecal microbiome	-	-	-
*L. rhamnosus*				
CLR2	Human fecal microbiome	-	-	-
ATCC 53103	Human fecal microbiome	-	+ +	-
*L. pentosus*				
BK1034	Olive oil	-	-	+
*L. plantarum*				
BK300	Olive oil	-	-	+
BK303	Olive oil	-	-	+
BK324	Olive oil	-	-	+
BK1135	Olive oil	-	-	+
BK1140	Olive oil	-	-	-
BK1039	Olive oil	-	-	+
BK1040	Olive oil	-	-	+
BK213	Olive oil	-	-	-
BK232	Olive oil	-	-	-
BK251	Olive oil	-	-	-
BK180	Olive oil	-	-	-
BK194	Olive oil	-	-	-
CHOL-200	Human fecal microbiome	+ +	+ +	-
*L. acidophilus*				
BK1197	Human oral mucosa	-	-	+
ATCC 314	Human	-	+ +	+
ATCC 4355	Rat	-	+ +	+
ATCC 4356	Human	-	+	-
ATCC 4796	Unknown	-	+	+
ATCC 53544	Human infant, rectal swab	-	+ +	+
ATCC 53671	Swine intestine	-	+ +	+
ATCC 9224	Unknown	-	+	-
LMG 11466	Unknown	-	-	+
CL1285	Human fecal microbiome	+ +	+ +	+ +
FERM BP-4980	Human fecal microbiome	-	-	+
FERM BP-4981	Human fecal microbiome	-	-	+
*L. crispatus*				
BK343	Human vaginal mucosa	-	-	+
BK482	Human vaginal mucosa	-	-	+
ATCC 55221	Derived from existing strain	-	+ +	-
*L. gallinarum*				
LMG 9435	Chicken crop	-	-	+
*L. gasseri*				
ATCC 33323	Human	-	-	+
*L. buchneri*				
BK308	Olive oil	-	-	-
BK287	Olive oil	-	-	-
BK235	Olive oil	-	-	-
BK170	Olive oil	-	-	-
BK187	Olive oil	-	-	-
BK192	Olive oil	-	-	-
BK1062	Olive oil	-	-	-
*L. brevis*				
BK107	Fermented food	-	-	-
BK115	Fermented food	-	-	-
BK205	Fermented food	-	-	-
*L. namurensis*				
BK298	Olive oil	-	-	-
BK302	Olive oil	-	-	-
BK317	Olive oil	-	-	-
BK330	Olive oil	-	-	-
BK334	Olive oil	-	-	-
BK270	Olive oil	-	-	-
BK214	Olive oil	-	-	+
*L. vaginalis*				
BK337	Human vaginal mucosa	+	-	-
BK372	Human vaginal mucosa	+	-	-
BK373	Human vaginal mucosa	+	-	+
BK349	Human vaginal mucosa	+	-	+
BK488	Human vaginal mucosa	+	-	+
BK383	Human vaginal mucosa	-	-	+
BK384	Human vaginal mucosa	-	-	-

^a^*GCA*, glycine-conjugated bile acid

^b^*TCA*, taurine-conjugated bile acid

**Fig. 1:**
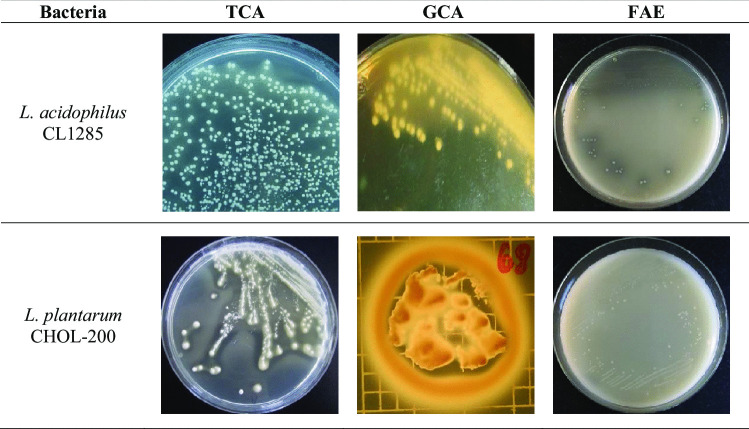
Visual representation of BSH- and FAE-positive activities of *L. acidophilus* CL1285 and *L. plantarum* CHOL-200. Strains were grown in the presence of TCA and GCA for 72 h under an anaerobic atmosphere at 37 °C or in the presence of ethyl ferulate (FAE) for up to 6 days under an anaerobic atmosphere at 37 °C. No FAE activity was detected for *L. plantarum* CHOL-200

In parallel, the same bacterial strains were also evaluated for FAE activity. Twenty-six strains were positive for FAE activity (Table 2): seven of genus *Lactiplantibacillus*, 14 of genus *Lactobacillus*, one from *Levilactobacillus* genus, and four of genus *Limosilactobacillus*. However, only one of these strains harbored strong FAE activity, *L. acidophilus* CL1285. Representative data of BSH- and FAE-positive activity is shown in Fig. 1, using *L. acidophilus* CL1285 and *L. plantarum* CHOL-200 results as reference.

**Fig. 2:**
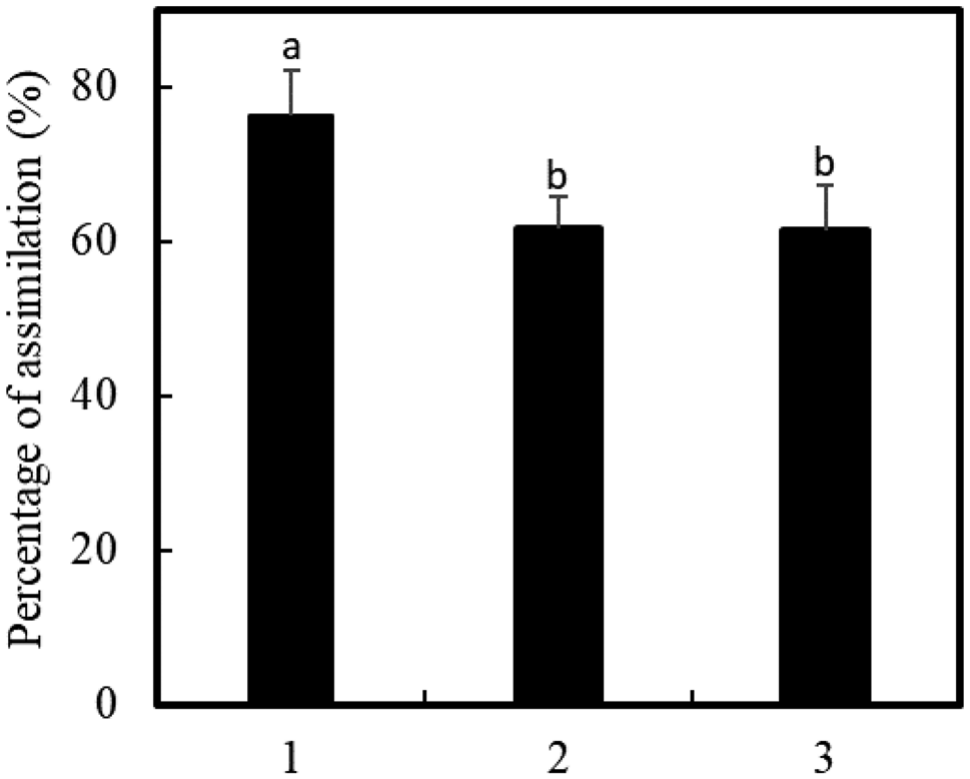
Percentage of cholesterol assimilation. 1: *L. plantarum* CHOL-200, 2: *L. acidophilus* CL1285, 3: *L. acidophilus* ATCC 43121. Numbers bearing a different letter are significantly different (*p* ≤ 0.05)

Following this screening approach, two candidates were selected for further characterization. *L. plantarum* CHOL-200 showed strong BSH activity for taurine- or glycine-conjugated bile acids and *L. acidophilus* CL1285 showed strong activity in both BSH and the FAE assays. Both strains were then assessed for their capacity to assimilate cholesterol, a third suggested mechanisms to explain the strain-specific capacity of some bacteria to decrease cholesterol in vivo. In addition to these two strains, cholesterol assimilation was measured for *L. acidophilus* ATCC 43121 as positive control [29, 31]. Both CHOL-200 and CL1285 demonstrated the ability to assimilate cholesterol present in the culture media. A cholesterol removal rate of 76.3% was observed for *L. plantarum* CHOL-200 and 61.5% for *L*. *acidophilus* CL1285. This is comparable to the cholesterol removal rate obtained with the positive control strain *L. acidophilus* ATCC 43121 (Fig. 2).

**Fig. 3:**
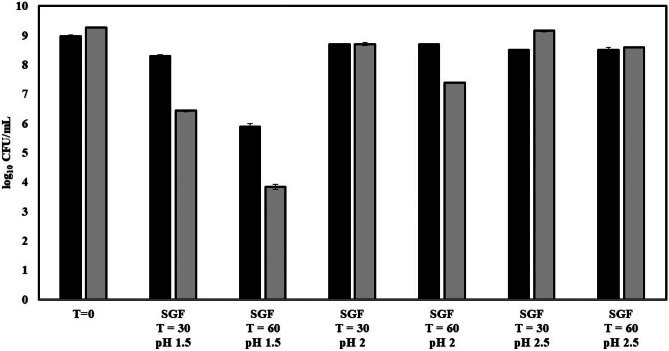
Gastric survival of *L. acidophilus* CL1285 and *L. plantarum* CHOL-200 when exposed to SGF of pH 1.5 to 2.5 for 30 and 60 min. Black bar: *L. acidophilus* CL1285, gray bar: *L. plantarum* CHOL-200

To exert their beneficial effects, probiotic bacteria must resist the harsh acidic and bilious environments of the stomach and small intestine, respectively. To further characterize the probiotic properties of *L. acidophilus* CL1285 and *L. plantarum* CHOL-200, both strains were challenged in SGF, simulating the acidic and enzymatic stomach conditions, of pH 1.5 to 2.5 for 30 and 60 min (Fig. 3). *L. acidophilus* CL1285 is unaffected (difference from the initial bacterial count < 1 Log_10_ CFU/mL) by up to 60 min of exposure to SGF pH 2 and 2.5. *L. acidophilus* CL1285 is also resistant to SGF at pH 1.5 for 30 min. However, viability was reduced by 3.07 Log_10_ CFU/mL after 60 min in SGF pH 1.5. *L. plantarum* CHOL-200 survived for up to 60 min in SGF pH 2.5 and for 30 min in SGF pH 2. However, decreased viability by nearly 1.9 Log_10_ CFU/mL was observed after 60 min in SGF pH 2. CHOL-200 was minimally resistant to SGF pH 1.5 for 30 or 60 min as shown by a reduction of 2.84 and 5.43 Log_10_ CFU/mL respectively.

Goldin and Gorbach state that probiotic bacteria should be able to grow in the presence of 0.15 to 0.3% bile salt [44]. *L. acidophilus* CL1285 and *L. plantarum* CHOL-200 were therefore evaluated for tolerance to three different commercial mixtures of bile acids (Table 3). CL1285 was able to grow in the presence of Ox gall powder, bacteriological bile, and 0.15% bile salts. However, an inhibitory effect on growth was observed in the presence of 0.3% bile salts. CHOL-200 grew on MRS supplemented with all three bile salts, regardless of the concentration tested. Of the three products tested, both strains showed a greater resistance to the presence of Ox gall powder. Hu et al. assessed the composition of different commercial ox gall powders and showed that the Ox gall powder from Sigma-Aldrich is similar to human bile based on the ratios of bile acids present. This observation supports the choice of Sigma-Aldrich Ox gall powder as the most appropriate to evaluate the bile resistance of probiotic bacteria [45].

**Table 3:** Growth of *L. acidophilus* CL1285 and *L. plantarum* CHOL-200, after 48 h under anaerobic atmosphere, on MRS supplemented with 0.15% and 0.3% of bile salts (w/v). -: no growth, + : growth, +  + : strong growth

Bacteria	MRS	Ox gall powder^a^		Bile salts^b^		Bacteriological bile^c^	
		**0.15%**	**0.3%**	**0.15%**	**0.3%**	**0.15%**	**0.3%**
CHOL-200	+ +	+ +	+ +	+ +	+	+ +	+ +
CL1285	+ +	+ +	+ +	+	-	+	+

^a^Bile bovine consisting of 70% bile salts, 22% phospholipids, 4% cholesterol, 3% proteins, and 0.3% bilirubin (source: Sigma-Aldrich)

^b^Mixture of ~ 50% cholic acid and ~ 50% deoxycholic acid (source: Sigma-Aldrich)

^c^Bile acids (as cholic acid) corresponding to ≥ 45% of dry matter (source: Organotechnie)

## Discussion

Elevated levels of TC, especially from LDL particles, are a major risk factor for the development of CVD. Although a number of therapeutic strategies are applied in order to manage hypercholesterolemia, only 32.1 to 55.7% of patients reach LDL-C targets providing a cardiovascular risk reduction [46]. In addition, side effects observed in some patients, with the use of statins for example, can lead to a decrease of adherence to treatment and therapy discontinuation [47]. Based on this, there is opportunity for new lipid-lowering natural products, to be used alone or in combination with actual proposed pharmaceutical treatments. Until now, numerous studies have evaluated the capacity of nutraceuticals, like berberine, green tea extracts, and probiotics, to impact blood lipid levels [6]. Clinical evidence showed the efficacy of some probiotic bacteria, especially strains related to *Lactobacillaceae*, to improve lipid profile [11]. In 2017, a group of experts stated that data available at the time did not allow recommending probiotics as an alternative treatment for managing dyslipidemia [6]. Nevertheless, probiotics are safe, with no side effects reported after consumption to date. The potential preventive effect of probiotics on dysregulated lipid profiles cannot be excluded and should be explored further based on studies on the complex interaction between the gut microbiome and digestive function. To fulfill the need for new, efficient, lipid-lowering probiotic formulations, candidate probiotic strains were selected with in vitro properties related to mechanisms that are proposed to result in the cholesterol-lowering effects of some bacteria. Among all suggested mechanisms, BSH and FAE activities were selected as screening targets.

Of 70 *Lactobacillaceae* strains tested against tauro-conjugated and glyco-conjugated cholic acids, 18 showed BSH activity for either one or the other form of bile salts or both. This is in agreement with other studies demonstrating that BSH are not ubiquitous among lactobacilli [48, 49] and that their substrate specificity varies greatly [50, 51]. In this study, most BSH-positive strains exhibited specificity for taurocholic acid. *L. acidophilus* CL1285 and *L. plantarum* CHOL-200, in addition to having the strongest BSH activity, were the only strains, besides *L. casei* LBC80R, to show in vitro activity toward both glycocholic acid and taurocholic acid. Although more analysis is needed to determine substrate preference, the capacity to hydrolyze bile acids conjugated to taurine and glycine could be beneficial for *L. acidophilus* CL1285 and *L. plantarum* CHOL-200 by providing them an adaptation advantage for the diverse pool of bile salts found in the human gut [50]. Thirteen strains of *L. plantarum* were tested on BSH agar plate assay. Interestingly, only CHOL-200 hydrolyzed bile salts. However, according to literature, many other *L. plantarum* strains harbor BSH activity [49]. Most of BSH-positive strains presented here have been isolated from fecal samples. This is in line with the importance of BSH in shaping the gut microbiota, for example, by allowing bacteria to resist the toxic effects of some bile acids in the gastrointestinal (GI) tract [50]. O’Flaherty et al. [49] showed that *Lactobacillus*-encoded BSH proteins were typically associated with the GI tract of vertebrates, suggesting a selective pressure to adapt to this environment.

Twenty-six strains were positive for FAE activity, with *L. acidophilus* CL1285 exhibiting the strongest. These strains were isolated from plants, animals, or humans which is consistent with functionality of microbial FAE releasing phenolic compounds, such as FA, from plant cell walls, thus supporting digestion of dietary fibers. Our results showed that not all *Lactobacillus* have the ability to produce FA via FAE activity. This is supported by a previous study by Liu et al. [52], where only 12 of the 33 strains tested were able to produce FAE activity. Once liberated, FA can act in many ways including as an antioxidant by scavenging reactive oxygen species (ROS), as natural antimicrobial compound, and as an anti-inflammatory agent, thus playing a major role as an anti-diabetic molecule and conferring many other desirable therapeutic benefits [53].

In addition to FAE and/or BSH activities, our results showed that *L. plantarum* CHOL-200 and *L. acidophilus* CL1285 have the ability to assimilate cholesterol in vitro, a characteristic widespread among lactobacilli [54]. Multiple mechanisms have been proposed for this ability. Cholesterol could be incorporated into the cellular membrane of the bacteria, bind to the surface of lactobacilli by adsorption, or be assimilated and transferred directly into the cytoplasm of the bacteria [30, 31, 55]. Both strains are also able to survive an in vitro simulated gastric environment and grow in the presence of bile acids, two essential properties that a probiotic should possess to survive passage through the GI tract and allow for exerting beneficial effects on the host [44].

Initial selection of BSH and FAE was based on evidence that this in vitro activity can translate to animal models and humans. In an in vivo study, a significant decrease in LDL-C and TC serum level was observed using fermented milk containing BSH-positive *L. casei* F0822 but no effect was found when BSH genes were removed [56]. Following in vitro screening of lactic acid bacteria for their capacity to deconjugate bile salts, remove cholesterol, and modulate NPC1L1 protein levels, only identification of BSH activity correlated with a lowering-cholesterol effect by *L. plantarum* CAAS 18008 in an animal model [57]. *L. plantarum* ECGC 13110402, a probiotic strain selected for its BSH activity, was shown to reduce TC and LDL-C in normal, mildly hypercholesterolemic and hypercholesterolemic individuals [58, 59]. Along the same lines, administration of FA extract was shown to reduce LDL-C in rats [27] and, more recently, to impact favorably and significantly TC, LDL-C, triglycerides, and HDL-C in hypercholesterolemic patients [60]*.* Supplementation with *L. fermentum* CRL1446, an FAE-positive bacteria, reduced triglycerides and TC levels in animal models [61]. Moreover, *L. fermentum* NCIMB 5221, another FAE-positive strain, significantly reduced triglycerides and LDL-C as well as atherogenic and atherosclerosis index in a rat model displaying hyperglycemia and hyperlipidemia [16]. Also, administration of a blend of *L. rhamnosus* NCIMB 6375, *L. plantarum* NCIMB 8826, and *L. fermentum* NCIMB 5221, combining the BSH activity of NCIMB 8826 and the FAE activity of NCIMB 5221, was shown to reduce TC, LDL-C, and triglyceride levels in an high fat (HF) diet hamster model [62].

Independent of the mechanism of the action proposed, numerous studies have shown that probiotic interventions have a beneficial impact on the blood lipid profiles [12, 13, 63, 64]. More specifically, subgroup analysis suggested that consumption of the probiotic strains *L. plantarum* or *L. reuteri* is particularly effective in reducing TC and LDL-C in hypercholesterolemic patients [12]. In other meta-analyses, probiotic formulations containing *L. acidophilus* showed significant reduction of TC and/or LDL-C [13, 63]. However, these meta-analyses were performed only on a limited number of specific strains, sometimes as part of multi-strain formulations, and found variable impacts on cholesterol levels, suggesting that cholesterol-lowering efficacy cannot be generalized at the species level.

The impact of *L. acidophilus* CL1285 and *L. plantarum* CHOL-200 on blood lipids alone or in combination has been evaluated in a hypercholesterolemic hamster model (unpublished data). Results demonstrated that *L. acidophilus* CL1285 tends to decrease the concentration of non-HDL-C (*p* = 0.08) with this effect becoming significant when *L. acidophilus* CL1285 is combined with *L. plantarum* CHOL-200 (*p* ≤ 0.05). In addition, the CL1285 strain alone and in combination with CHOL-200 significantly increased the HDL:cholesterol ratio (*p* ≤ 0.05). Impacts on non-HDL-C concentration and HDL:cholesterol ratio seem to be driven by *L. acidophilus* CL1285 as administration of *L. plantarum* CHOL-200 showed opposite effects. However, more analysis is needed to decipher the exact mechanism at work, whether BSH, FAE, a combination of both activities, or even the capacity to assimilate cholesterol is responsible in vivo for the improvement in lipid profile observed with *L. acidophilus* CL1285 and the combination of both CL1258 and CHOL-200.

In summary, an approach targeting multiple bacterial lipid-lowering mechanisms, BSH and FAE activity, has led to the selection of *L. acidophilus* CL1285 and *L. plantarum* CHOL-200. Both strains exhibited strong abilities, relatively to their probiotic properties and their multiple cholesterol-lowering mechanisms, suggesting they can be considered good probiotic candidates for the management of dyslipidemia. In addition, their mechanisms of action suggest they could harbor the capacity to act on various therapeutic targets in vivo. An in vivo assay, using a hypercholesterolemic hamster model, demonstrated the ability of *L. acidophilus* CL1285 alone and in combination with *L. plantarum* CHOL-200, but not of *L. plantarum* CHOL-200 alone, to reduce blood non-HDL-C concentration and to improve HDL:cholesterol ratio. However, more work will be needed to validate the exact mechanism of action supporting the cholesterol-lowering effect observed in this study and to determine if these results can be translated to humans.

## Data Availability

Data sharing is not applicable to this article as no datasets were generated or analyzed during the current study.

## References

[1] G. B. D. Causes of Death Collaborators,  (2018). Global, regional, and national age-sex-specific mortality for 282 causes of death in 195 countries and territories, 1980–2017: a systematic analysis for the Global Burden of Disease Study 2017. Lancet.

[2] Imamura T, Doi Y, Arima H, Yonemoto K, Hata J, Kubo M, Tanizaki Y, Ibayashi S, Iida M, Kiyohara Y (2009) LDL cholesterol and the development of stroke subtypes and coronary heart disease in a general Japanese population: the Hisayama study. Stroke 40:382–388. 10.1161/STROKEAHA.108.529537 10.1161/STROKEAHA.108.529537 19095987

[3] Stamler J, Neaton JD (2008). The multiple risk factor intervention trial (MRFIT)–importance then and now. JAMA.

[4] Ramkumar S, Raghunath A, Raghunath S (2016). Statin therapy: review of safety and potential side effects. Acta Cardiol Sin.

[5] Davies JT, Delfino SF, Feinberg CE, Johnson MF, Nappi VL, Olinger JT, Schwab AP, Swanson HI (2016). Current and emerging uses of statins in clinical therapeutics: a review. Lipid Insights.

[6] Cicero AFG, Colletti A, Bajraktari G, Descamps O, Djuric DM, Ezhov M, Fras Z, Katsiki N, Langlois M, Latkovskis G, Panagiotakos DB, Paragh G, Mikhailidis DP, Mitchenko O, Paulweber B, Pella D, Pitsavos C, Reiner Z, Ray KK, Rizzo M, Sahebkar A, Serban MC, Sperling LS, Toth PP, Vinereanu D, Vrablik M, Wong ND, Banach M (2017). Lipid lowering nutraceuticals in clinical practice: position paper from an International Lipid Expert Panel. Arch Med Sci.

[7] Yeh GY, Davis RB, Phillips RS (2006). Use of complementary therapies in patients with cardiovascular disease. Am J Cardiol.

[8] Hunter PM, Hegele RA (2017). Functional foods and dietary supplements for the management of dyslipidaemia. Nat Rev Endocrinol.

[9] Hill C, Guarner F, Reid G, Gibson GR, Merenstein DJ, Pot B, Morelli L, Canani RB, Flint HJ, Salminen S, Calder PC, Sanders ME (2014). Expert consensus document. The International Scientific Association for Probiotics and Prebiotics consensus statement on the scope and appropriate use of the term probiotic. Nat Rev Gastroenterol Hepatol.

[10] Miremadi F, Ayyash M, Sherkat F, Stojanovska L (2014) Cholesterol reduction mechanisms and fatty acid composition of cellular membranes of probiotic Lactobacilli and Bifidobacteria. J Funct Foods 9:295–305. 10.1016/j.jff.2014.05.002

[11] Miremadi F, Sherkat F, Stojanovska L (2016) Hypocholesterolaemic effect and anti-hypertensive properties of probiotics and prebiotics: a review. J Funct Foods 25:497–510. 10.1016/j.jff.2016.06.016

[12] Wu Y, Zhang Q, Ren Y, Ruan Z (2017). Effect of probiotic *Lactobacillus* on lipid profile: a systematic review and meta-analysis of randomized, controlled trials. PLoS ONE.

[13] Sun J, Buys N (2015). Effects of probiotics consumption on lowering lipids and CVD risk factors: a systematic review and meta-analysis of randomized controlled trials. Ann Med.

[14] Huang Y, Zheng Y (2010). The probiotic *Lactobacillus acidophilus* reduces cholesterol absorption through the down-regulation of Niemann-Pick C1-like 1 in Caco-2 cells. Br J Nutr.

[15] Ishimwe N, Daliri EB, Lee BH, Fang F, Du G (2015). The perspective on cholesterol-lowering mechanisms of probiotics. Mol Nutr Food Res.

[16] Tomaro-Duchesneau C, Saha S, Malhotra M, Jones ML, Labbe A, Rodes L, Kahouli I, Prakash S (2014). Effect of orally administered *L. fermentum* NCIMB 5221 on markers of metabolic syndrome: an in vivo analysis using ZDF rats. Appl Microbiol Biotechnol.

[17] Bateup JM, McConnell MA, Jenkinson HF, Tannock GW (1995). Comparison of *Lactobacillus* strains with respect to bile salt hydrolase activity, colonization of the gastrointestinal tract, and growth rate of the murine host. Appl Environ Microbiol.

[18] Coleman JP, Hudson LL (1995). Cloning and characterization of a conjugated bile acid hydrolase gene from *Clostridium perfringens*. Appl Environ Microbiol.

[19] Franz CM, Specht I, Haberer P, Holzapfel WH (2001). Bile salt hydrolase activity of Enterococci isolated from food: screening and quantitative determination. J Food Prot.

[20] Grill JP, Manginot-Durr C, Schneider F, Ballongue J (1995). Bifidobacteria and probiotic effects: action of *Bifidobacterium* species on conjugated bile salts. Curr Microbiol.

[21] Kawamoto K, Horibe I, Uchida K (1989). Purification and characterization of a new hydrolase for conjugated bile acids, chenodeoxycholyltaurine hydrolase, from *Bacteroides vulgatus*. J Biochem.

[22] Liong MT, Shah NP (2005) Bile salt deconjugation ability, bile salt hydrolase activity and cholesterol co-precipitation ability of lactobacilli strains. Int Dairy J 15:391–398. 10.1016/j.idairyj.2004.08.007

[23] Pereira DI, Gibson GR (2002). Effects of consumption of probiotics and prebiotics on serum lipid levels in humans. Crit Rev Biochem Mol Biol.

[24] Benoit I, Danchin EG, Bleichrodt RJ, de Vries RP (2008). Biotechnological applications and potential of fungal feruloyl esterases based on prevalence, classification and biochemical diversity. Biotechnol Lett.

[25] Esteban-Torres M, Reveron I, Mancheno JM, de Las RB, Munoz R (2013). Characterization of a feruloyl esterase from *Lactobacillus plantarum*. Appl Environ Microbiol.

[26] Tomaro-Duchesneau C, Saha S, Malhotra M, Coussa-Charley M, Al-Salami H, Jones M, Labbe A, Prakash S (2012). *Lactobacillus fermentum* NCIMB 5221 has a greater ferulic acid production compared to other ferulic acid esterase producing Lactobacilli. Int J Probiotics Prebiotics.

[27] Kim HK, Jeong TS, Lee MK, Park YB, Choi MS (2003). Lipid-lowering efficacy of hesperetin metabolites in high-cholesterol fed rats. Clin Chim Acta.

[28] Dominguez-Avila JA, Gonzalez-Aguilar GA, Alvarez-Parrilla E, de la Rosa LA (2016). Modulation of PPAR expression and activity in response to polyphenolic compounds in high fat diets. Int J Mol Sci.

[29] Gilliland SE, Nelson CR, Maxwell C (1985). Assimilation of cholesterol by *Lactobacillus acidophilus*. Appl Environ Microbiol.

[30] Lye H-S, Rusul G, Liong MT (2010). Removal of cholesterol by lactobacilli via incorporation and conversion to coprostanol. J Dairy Sci.

[31] Noh DO, Kim SH, Gilliland SE (1997) Incorporation of cholesterol into the cellular membrane of *Lactobacillus acidophilus* ATCC 431211. J Dairy Sci 80:3107–3113. 10.3168/jds.S0022-0302(97)76281-7 10.3168/jds.S0022-0302(97)76281-7 9436091

[32] Gao XW, Mubasher M, Fang CY, Reifer C, Miller LE (2010). Dose-response efficacy of a proprietary probiotic formula of *Lactobacillus acidophilus* CL1285 and *Lactobacillus casei* LBC80R for antibiotic-associated diarrhea and *Clostridium difficile*-associated diarrhea prophylaxis in adult patients. Am J Gastroenterol.

[33] Kullar R, Johnson S, McFarland LV, Goff DA, Goldstein EJC (2020). Bundling probiotics with antimicrobial stewardship programs for the prevention of *Clostridiodes difficile* infections in acute care hospitals. Infect Dis Clin Pract.

[34] McFarland LV, Ship N, Auclair J, Millette M (2018). Primary prevention of *Clostridium difficile* infections with a specific probiotic combining *Lactobacillus acidophilus*, *L. casei*, and *L. rhamnosus* strains: assessing the evidence. J Hosp Infect.

[35] Preston K, Krumian R, Hattner J, de Montigny D, Stewart M, Gaddam S (2018). *Lactobacillus acidophilus* CL1285, *Lactobacillus casei* LBC80R and *Lactobacillus rhamnosus* CLR2 improve quality-of-life and IBS symptoms: a double-blind, randomised, placebo-controlled study. Benef Microbes.

[36] Ship N, Mallais C, Carrière S (2019) Burden of IBS-diarrhea symptoms tracked with daily journals for 12 weeks in a randomized, double-blind, placebo-controlled study of *Lactobacillus acidophilus* CL1285, *L. casei* LBC80R and *L. rhamnosus* CLR2. Am J Gastroenterol 114:p S296. 10.14309/01.ajg.0000591568.36430.d7

[37] Edwards U, Rogall T, Blöcker H, Emde M, Böttger EC (1989). Isolation and direct complete nucleotide determination of entire genes. Characterization of a gene coding for 16S ribosomal RNA. Nucleic Acids Res.

[38] Aroutcheva A, Auclair J, Frappier M, Millette M, Lolans K, de Montigny D, Carriere S, Sokalski S, Trick WE, Weinstein RA (2016). Importance of molecular methods to determine whether a probiotic is the source of *Lactobacillus* bacteremia. Probiotics Antimicrob Proteins.

[39] Pereira DI, McCartney AL, Gibson GR (2003). An in vitro study of the probiotic potential of a bile-salt-hydrolyzing *Lactobacillus fermentum* strain, and determination of its cholesterol-lowering properties. Appl Environ Microbiol.

[40] Donaghy J, Kelly PF, McKay AM (1998). Detection of ferulic acid esterase production by *Bacillus* spp. and lactobacilli. Appl Microbiol Biotechnol.

[41] Razin S, Kutner S, Efrati H, Rottem S (1980). Phospholipid and cholesterol uptake by *Mycoplasma* cells and membranes. Biochim Biophys Acta.

[42] Rudel LL, Morris MD (1973). Determination of cholesterol using o-phthalaldehyde. J Lipid Res.

[43] Corp. I, IBM Corp., Armonk, NY Released 2010.

[44] Goldin BR, Gorbach SL (1992) Probiotics for humans. In: Probiotics. Dordrecht, pp 355–376

[45] Hu PL, Yuan YH, Yue TL, Guo CF (2018). Bile acid patterns in commercially available oxgall powders used for the evaluation of the bile tolerance ability of potential probiotics. PLoS ONE.

[46] Danchin N, Almahmeed W, Al-Rasadi K, Azuri J, Berrah A, Cuneo CA, Karpov Y, Kaul U, Kayikcioglu M, Mitchenko O, Ruiz AJ, Aguilar Salinas CA, Santos RD, Mercier F, Blom D, Investigators I (2018). Achievement of low-density lipoprotein cholesterol goals in 18 countries outside Western Europe: The International ChoLesterol management Practice Study (ICLPS). Eur J Prev Cardiol.

[47] Maningat P, Gordon BR, Breslow JL (2013). How do we improve patient compliance and adherence to long-term statin therapy?. Curr Atheroscler Rep.

[48] Liang L, Yi Y, Lv Y, Qian J, Lei X, Zhang G (2018). A Comprehensive Genome Survey Provides Novel Insights into Bile Salt Hydrolase (BSH) in *Lactobacillaceae*. Molecules.

[49] O'Flaherty S, Briner Crawley A, Theriot CM, Barrangou R (2018) The *Lactobacillus* bile salt hydrolase repertoire reveals niche-specific adaptation. mSphere 3. 10.1128/mSphere.00140-18 10.1128/mSphere.00140-18 PMC5976879 29848760

[50] Foley MH, O'Flaherty S, Allen G, Rivera AJ, Stewart AK, Barrangou R, Theriot CM (2021). *Lactobacillus* bile salt hydrolase substrate specificity governs bacterial fitness and host colonization. Proc Natl Acad Sci U S A.

[51] Jiang J, Hang X, Zhang M, Liu X, Li D, Yang H (2010). Diversity of bile salt hydrolase activities in different lactobacilli toward human bile salts. Ann Microbiol.

[52] Liu S, Bischoff KM, Anderson AM, Rich JO (2016). Novel feruloyl esterase from *Lactobacillus fermentum* NRRL B-1932 and analysis of the recombinant enzyme produced in *Escherichia coli*. Appl Environ Microbiol.

[53] Paiva LBd, Goldbeck R, Santos WDd, Squina FM (2013). Ferulic acid and derivatives: molecules with potential application in the pharmaceutical field. Braz J Pharm Sci.

[54] Albano C, Morandi S, Silvetti T, Casiraghi MC, Manini F, Brasca M (2018). Lactic acid bacteria with cholesterol-lowering properties for dairy applications: in vitro and in situ activity. J Dairy Sci.

[55] Iranmanesh M, Ezzatpanah H, Mojgani N (2014). Antibacterial activity and cholesterol assimilation of lactic acid bacteria isolated from traditional Iranian dairy products. LWT - Food Sci Technol.

[56] Guo CF, Zhang S, Yuan YH, Li JY, Yue TL (2018). Bile salt hydrolase and S-layer protein are the key factors affecting the hypocholesterolemic activity of *Lactobacillus casei*-fermented milk in Hamsters. Mol Nutr Food Res.

[57] Ma C, Zhang S, Lu J, Zhang C, Pang X, Lv J (2019). Screening for cholesterol-lowering probiotics from lactic acid bacteria isolated from corn silage based on three hypothesized pathways. Int J Mol Sci.

[58] Costabile A, Buttarazzi I, Kolida S, Quercia S, Baldini J, Swann JR, Brigidi P, Gibson GR (2017). An *in vivo* assessment of the cholesterol-lowering efficacy of *Lactobacillus plantarum* ECGC 13110402 in normal to mildly hypercholesterolaemic adults. PLoS ONE.

[59] Keleszade E, Kolida S, Costabile A (2022) The cholesterol lowering efficacy of *Lactobacillus plantarum* ECGC 13110402 in hypercholesterolemic adults: a double-blind, randomized, placebo controlled, pilot human intervention study. J Funct Foods 89:104939. 10.1016/j.jff.2022.104939

[60] Bumrungpert A, Lilitchan S, Tuntipopipat S, Tirawanchai N, Komindr S (2018). Ferulic acid supplementation improves lipid profiles, oxidative stress, and inflammatory status in hyperlipidemic subjects: a randomized, double-blind, placebo-controlled clinical trial. Nutrients.

[61] Russo M, Fabersani E, Abeijon-Mukdsi MC, Ross R, Fontana C, Benitez-Paez A, Gauffin-Cano P, Medina RB (2016). *Lactobacillus fermentum* CRL1446 ameliorates oxidative and metabolic parameters by increasing intestinal feruloyl esterase activity and modulating microbiota in caloric-restricted mice. Nutrients.

[62] Iqbal UH, Westfall S, Prakash S (2018). Novel microencapsulated probiotic blend for use in metabolic syndrome: design and *in-vivo* analysis. Artif Cells Nanomed Biotechnol.

[63] Shimizu M, Hashiguchi M, Shiga T, Tamura HO, Mochizuki M (2015). Meta-analysis: effects of probiotic supplementation on lipid profiles in normal to mildly hypercholesterolemic individuals. PLoS ONE.

[64] Wang L, Guo MJ, Gao Q, Yang JF, Yang L, Pang XL, Jiang XJ (2018). The effects of probiotics on total cholesterol: a meta-analysis of randomized controlled trials. Medicine (Baltimore).

